# Association between body mass index and long-term all-cause mortality in critically ill patients without malignant tumors

**DOI:** 10.1371/journal.pone.0325452

**Published:** 2025-06-25

**Authors:** Jian Deng, Linyan Zhao

**Affiliations:** Department of Critical Care Medicine, The Second Hospital of Dalian Medical University, Dalian, Liaoning, China; Tehran University of Medical Sciences, IRAN, ISLAMIC REPUBLIC OF

## Abstract

**Background:**

The “obesity paradox” in certain diseases has been reported in previous studies. This study aimed to investigate the relationship between BMI and long-term mortality in all critically ill patients without malignant tumors who were admitted to the ICU.

**Methods:**

Using the MIMIC-IV 2.2 database, we included all ICU admissions for patients without malignant tumors and categorized them into four groups based on the World Health Organization (WHO) obesity criteria. The relationship between BMI and 90-day, 180-day, and 1-year mortality was analyzed using univariate and multivariate Cox regression models, along with restricted cubic spline (RCS) models to account for potential non-linear associations.

**Results:**

A total of 19,089 patients were included, with 90-day, 180-day, and 1-year mortality rates of 18.35%, 20.80%, and 23.96%, respectively. Overweight and obese patients exhibited significantly lower mortality rates compared to underweight and normal-weight individuals at all time points. After adjusting for confounders, higher BMI remained a protective factor for long-term mortality (HR 0.65–0.72, *P* < 0.001). RCS curves demonstrated a U-shaped relationship between BMI and mortality, and subgroup analyses confirmed the protective effect of higher BMI in different subgroups.

**Conclusion:**

The “obesity paradox” may apply to critically ill patients without malignant tumors.

## Introduction

With rising living standards and shifting dietary patterns, nearly one-third of the global population now meets the WHO criteria for being overweight or obese [1, 2], Obesity has long been thought to be associated with an increased risk of numerous diseases, including hypertension, type 2 diabetes, coronary artery disease, stroke, and various cancers [3–6]. Traditionally, higher body mass index (BMI) is considered a negative predictor for health outcomes and mortality. However, recent studies have increasingly identified a paradoxical phenomenon in certain conditions, where individuals with a higher BMI exhibit a survival advantage compared to those with normal body weight. This intriguing observation is referred to as the “obesity paradox” [7, 8].

Several studies have explored the relationship between BMI and cause-specific prognosis [9–12], but whether the applicability of this phenomenon can be extended to the population of critically ill patients without malignant tumors, especially for long-term prognosis, is currently inconclusive.

Critically ill patients, often burdened with complex comorbidities and varying degrees of physiological stress [13], represent a unique population for studying the relationship between BMI and long-term outcomes. Unlike studies focusing on specific disease groups, our research aims to explore the association between BMI and one-year all-cause mortality in critically ill patients, specifically excluding those with malignant tumors, which is known to significantly alter metabolism and survival patterns [14]. The inclusion of a wide range of non-cancer critical patients allows for a more comprehensive analysis of BMI’s role in survival, potentially offering new insights into how obesity impacts outcomes in this highly vulnerable group.

## Methods

### Data source

This study utilized data from the Medical Information Mart for Intensive Care (MIMIC-IV) version 2.2, a large, open-access, single-center database containing information on over 70,000 adult patients admitted to the intensive care units (ICUs) at Beth Israel Deaconess Medical Center in Boston, Massachusetts, USA, between 2008 and 2019 [15]. Unlike the previous version, MIMIC-IV 2.2 offers a maximum follow-up time of one year after discharge. As all patient data were de-identified, the requirement for informed consent was waived. One author gained access to the database after successfully completing the Collaborative Institutional Training Initiative exam (certification number: 61993882).

### Study population and criteria

**Fig 1:**
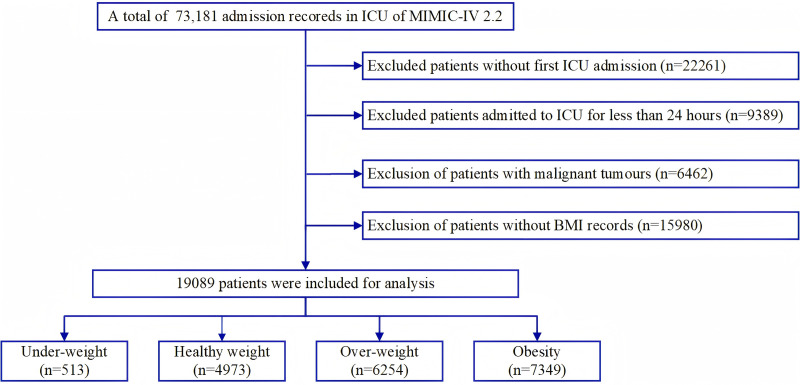
Flow chart of the participant selection.

Patients aged 18 years and older with complete BMI data were included in this study (Fig 1). For patients with multiple ICU admissions, only data from the first admission were used for analysis. The exclusion criteria were as follows: (1) ICU stays of less than 24 hours, and (2) patients with malignant tumors, including those with malignancies of the lungs, gastrointestinal tract, urinary tract, bone and bone marrow, skin, breast, and others.

### Statistical analysis

All patients were categorized into four groups based on the World Health Organization (WHO) BMI classification: underweight (BMI < 18.5 kg/m²), normal weight (18.5 ≤ BMI < 25 kg/m²), overweight (25 ≤ BMI < 30 kg/m²), and obese (BMI ≥ 30 kg/m²).

Statistical analysis was conducted using R 4.3.0. Continuous data with skewed distributions were expressed as median (Q₁, Q₃) and compared using the rank-sum test for multiple samples. Categorical data were expressed as n (%) and compared using the chi-square test or Fisher’s exact test, as appropriate. A p-value < 0.05 was considered statistically significant for between-group comparisons.

Kaplan-Meier survival curves were plotted for each BMI group to analyze the association between BMI as a categorical variable and 90-day, 180-day, and 1-year mortality rates. Differences between groups were assessed using the log-rank test. Adjusted hazard ratios (HRs) and 95% confidence intervals (CIs) were calculated using univariate and multivariate Cox proportional hazards regression models to assess the effect of predictors on mortality at 90 days, 180 days, and 1 year in critically ill patients.

Variables significant in univariate analysis (p < 0.05) were included in a multivariate Cox model to identify independent predictors of 1-year mortality. Clinically important variables, such as age, sex, SOFA score, and key comorbidities (e.g., heart failure, hypertension, type 2 diabetes mellitus), were also included in the multivariate model, regardless of statistical significance, to adjust for potential confounders. The normal-weight group (18.5 ≤ BMI < 25 kg/m²) was used as the reference category for the BMI variable.

A restricted cubic spline model with 4-knots was applied to a multivariate Cox proportional hazards regression model to examine the association between BMI as a continuous variable and 1-year mortality in critically ill patients. Subgroup analyses were performed to further explore the effect of BMI on 1-year mortality, stratified by specific etiologies of interest from previous studies. A p-value < 0.05 was considered statistically significant.

### Outcome

The primary outcome of this study was 1-year mortality, with secondary outcomes including 90-day and 180-day mortality.

### Data extraction

Data management was conducted using PostgreSQL. Extracted variables included demographic information, vital signs, laboratory results, comorbidities, treatment interventions, and severity scores, all recorded at the patient’s initial ICU admission. Demographic data comprised age and gender, while vital signs included mean blood pressure (MBP), heart rate (HR), respiratory rate (RR), body temperature, and oxygen saturation (SpO₂). Laboratory values included white blood cell count (WBC), hemoglobin (HGB), platelet count (PLT), serum sodium, potassium, chloride, Anion Gap and creatinine (Scr). Comorbidities such as congestive heart failure (CHF), hypertension, type 2 diabetes mellitus, myocardial infarction, chronic kidney disease (CKD), and acute renal failure(ARF) were also extracted. Scores reflecting the severity of the patient’s disease, including Sequential Organ Failure Assessment (SOFA) score, Oxford Acute Severity of Illness Score (OASIS), Acute Physiology Score III (APS III), Simplified Acute Physiology Score II (SAPS II), and Glasgow Coma Scale (GCS) were collected. Additionally, treatment interventions, such as renal replacement therapy (RRT), invasive mechanical ventilation (MV), and glucocorticoid use, were recorded. BMI was calculated as weight (kg) divided by height squared (m²). Missing data for continuous variables were less than 5% and were imputed using multiple imputation through the “MICE” package in R. The binary variables were complete, with no missing values identified.

## Results

A total of 19,089 eligible patients were included in the analysis (Table 1). Patients were categorized into four groups: 513 (2.68%) in the underweight group, 4,973 (26.05%) in the normal weight group, 6,254 (32.76%) in the overweight group, and 7,349 (38.49%) in the obese group.

**Table 1:** Baseline and clinical characteristics of the study patients by BMI categories.

Variables	Total (n = 19089)	Q1 (n = 513)	Q2 (n = 4973)	Q3 (n = 6254)	Q4 (n = 7349)	*P*
Age, years	66 (55, 76)	69 (54, 82)	68 (54, 80)	67 (56, 77)	64 (54, 73)	<.001
Male, n(%)	11622 (60.8)	222 (43.2)	2826 (56.8)	4159 (66.5)	4415 (60.0)	<.001
**Vital signs**						
HR	84 (74, 98)	88 (76, 103)	85 (74, 99)	83 (74, 96)	84 (75, 97)	<.001
MBP	78 (68, 90)	79 (67, 92)	78 (68, 91)	77 (68, 90)	78 (68, 90)	0.003
RR	17 (14, 22)	19 (16, 23)	17 (14, 22)	17 (14, 21)	17 (14, 21)	<.001
SPO2	99 (96, 100)	99 (96, 100)	99 (97, 100)	99 (96, 100)	99 (96, 100)	<.001
Temperature	36.7 (36.4, 37.0)	36.67 (36.3, 37.0)	36.7 (36.3, 37.0)	36.7 (36.4, 37.0)	36.7 (36.5, 37.1)	<.001
**Laboratory tests**						
WBC	11.4 (8.2, 15.5)	10.4 (7.4, 14.6)	10.7 (7.7, 14.8)	11.2 (8.2, 15.1)	12.1 (8.8, 16.3)	<.001
Platelet	177 (130, 237)	211 (151, 297)	182 (127, 245)	170 (125, 227)	179 (135, 236)	<.001
Hemoglobin	10.4 (8.9, 12.0)	10.3 (8.7, 11.5)	10.3 (8.8, 11.9)	10.5 (9.0, 12.1)	10.5 (9.0, 12.1)	<.001
Sodium	139 (136, 141)	139 (136, 142)	139 (136, 141)	139 (136, 141)	139 (136, 141)	0.713
Potassium	4.2 (3.8, 4.6)	4.1 (3.6, 4.5)	4.1 (3.7, 4.5)	4.2 (3.8, 4.6)	4.2 (3.9, 4.7)	<.001
Calcium	8.2 (7.9, 8.7)	8.3 (7.8, 8.8)	8.2 (7.8, 8.7)	8.2 (7.9, 8.7)	8.3 (7.9, 8.7)	0.010
Chloride	106 (102, 109)	104 (100, 108)	106 (101, 109)	106 (102, 110)	106 (102, 109)	<.001
Anion Gap	13 (11, 16)	14 (11, 17)	14 (11, 16)	13 (11, 16)	13 (11, 16)	<.001
pH	7.37 (7.34, 7.42)	7.37 (7.33, 7.42)	7.37 (7.35, 7.43)	7.37 (7.34, 7.42)	7.37 (7.33, 7.42)	<.001
Creatinine	0.9 (0.7, 1.3)	0.8 (0.6, 1.3)	0.9 (0.7, 1.3)	0.9 (0.7, 1.3)	1.0 (0.8, 1.4)	<.001
**Scoring system**						
SOFA	5 (2, 5)	4 (2, 5)	4 (2, 5)	5 (2, 5)	5 (3,6)	<.001
APSIII	40 (12, 56)	46 (0, 60)	41 (13, 56)	38 (14, 54)	39 (12, 56)	<.001
SAPSII	35 (14, 45)	36 (14, 46)	35 (14, 45)	35 (14, 44)	36 (14, 45)	0.654
Oasis	32 (15, 0)	34 (15, 0)	32 (15, 0)	32 (15, 0)	32 (15, 0)	0.004
GCS	15 (10, 11)	15 (9, 11)	15 (10, 11)	15 (10, 11)	15 (10, 11)	<.001
CCI	4 (2, 4)	5 (3, 4)	5 (2, 5)	4 (2, 4)	4 (2, 4)	<.001
**Comorbidities, n (%)**						
Hypertension	8684 (45.49)	169 (32.94)	1907 (38.35)	2904 (46.43)	3704 (50.40)	<.001
T2DM	5387 (28.22)	67 (13.06)	896 (18.02)	1581 (25.28)	2843 (38.69)	<.001
CHF	5111 (26.77)	117 (22.81)	1292 (25.98)	1608 (25.71)	2094 (28.49)	<.001
MI	1660 (8.70)	43 (8.38)	396 (7.96)	569 (9.10)	652 (8.87)	0.173
CKD	3082 (16.15)	68 (13.26)	728 (14.64)	1004 (16.05)	1282 (17.44)	<.001
ARF	5685 (29.78)	149 (29.04)	1325 (26.64)	1766 (28.24)	2445 (33.27)	<.001
**Interventions, n(%)**						
MV	11311 (59.25)	253 (49.32)	2710 (54.49)	3707 (59.27)	4641 (63.15)	<.001
RRT	1007 (5.28)	19 (3.70)	176 (3.54)	285 (4.56)	527 (7.17)	<.001
Glucocorticoids	3815 (19.99)	133 (25.93)	1143 (22.98)	1148 (18.36)	1391 (18.93)	<.001

Note: Q1: BMI < 18.5 kg/m^2^, Q2: 18.5 ≤ BMI < 25 kg/m², Q3: 25 ≤ BMI < 30 kg/m², Q4:BMI ≥ 30 kg/m²; HR, heart rate; MBP, mean blood pressure; RR, respiratory rate; SPO_2_, saturation of pulse oximetry; WBC, white blood cell; Ph, power of hydrogen; SOFA, sequential organ failure assessment; APS III, Acute Physiology Score III rating; SAPS Ⅱ, simplified acute physiology score Ⅱ; Oasisi, Oxford acute severity of illness score; CCI, Charlson comorbidity index; GCS, Glasgow coma scale; T2DM, diabetes mellitus type 2; CHF, congestive heart failure; MI, myocardial infarction; CKD, chronic kidney disease; ARF, acute renal failure, MV, mechanical ventilation; RRT, renal replacement therapy.

The analysis revealed that the overweight and obese groups tended to be older, and the higher BMI categories had a greater proportion of male patients. Patients in the higher BMI groups also had a higher likelihood of receiving mechanical ventilation and renal replacement therapy. Moreover, increased BMI was associated with a higher prevalence of comorbidities. The results of univariate COX regression and multivariate COX regression for all variables are presented in S1 Table.

### BMI and outcomes

Table 2 presents the outcomes for all study participants, revealing significant differences in ICU, in-hospital, 90-day, 180-day, and 1-year mortality rates across different BMI categories. The underweight group had the highest mortality rates, while mortality decreased progressively with increasing BMI. The obese group exhibited the lowest 90-day, 180-day, and 1-year mortality rates. The overweight group had the shortest ICU stay and overall hospitalization duration. All differences were statistically significant (P < 0.05).

**Table 2:** Outcomes of patients based on BMI categories.

Outcomes	Total	Q1	Q2	Q3	Q4	*P-value*
**(n = 19089)**	**(n = 513)**	**(n = 4973)**	**(n = 6254)**	**(n = 7349)**
ICU mortality, n (%)	1757 (9.20)	77 (15.01)	509 (10.24)	532 (8.51)	639 (8.70)	<.001
In-hospital mortality, n (%)	2357 (12.35)	111 (21.64)	729 (14.66)	698 (11.16)	819 (11.14)	<.001
90-day mortality, n (%)	3503 (18.35)	178 (34.70)	1171 (23.55)	1045 (16.71)	1109 (15.09)	<.001
180-day mortality, n (%)	3971 (20.80)	204 (39.77)	1334 (26.82)	1172 (18.74)	1261 (17.16)	<.001
1-year mortality, n (%)	4573 (23.96)	232 (45.22)	1519 (30.54)	1350 (21.59)	1472 (20.03)	<.001
ICU length of stay(days)	2.8 (1.5, 5.7)	3.1 (1.7, 5.7)	2.9 (1.7, 5.8)	2.6 (1.5, 5.2)	2.9 (1.5, 6.0)	<.001
Hospital length of stay(days)	7.9 (5.1, 13.7)	8.6 (4.9, 15.9)	8.0 (5.0, 14.0)	7.6 (5.0, 12.8)	8.2 (5.3, 13.9)	<.001

**Fig 2:**
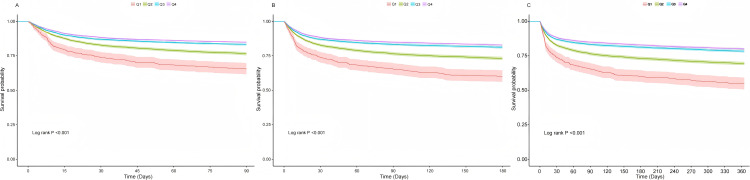
Kaplan-Meier curve for mortality by BMI category.

Kaplan-Meier analyses further demonstrated the impact of BMI on 90-day, 180-day, and 1-year mortality. A higher BMI was consistently associated with a lower risk of death across all time periods, with log-rank tests showing highly significant results (p < 0.001). The Fig 2 depict the survival analyses: (A. 90-day mortality survival analysis B. 180 mortality survival analysis C. 1-year mortality survival analysis).

Univariate and multivariate Cox regression analyses were conducted using BMI as a categorical variable, with the normal weight group serving as the reference. Prior to performing the multivariate Cox regression, the multicollinearity of covariates was assessed using the variance inflation factor (VIF). The results indicated that the Simplified Acute Physiology Score II (SAPS II) had a VIF value greater than 5, suggesting strong collinearity with other severity scores, such as the SOFA and APS III scores. To mitigate the impact of multicollinearity on model estimation, SAPS II was excluded from the final multivariate Cox regression model. After excluding this variable, no significant collinearity was detected among the remaining covariates (S2 Table).

In the adjusted Cox model (Table 3), underweight status was associated with an increased risk of death in 1-year, while both overweight and obesity were identified as protective factors when compared to the normal weight group, the same trend was observed in 90- and 180-day mortality rates.

**Table 3:** Multifactorial Cox regression analysis of 1-year mortality after adjustment for all covariates.

BMI levels	Model Ⅰ		Model Ⅱ	
HR (95%CI)	*P-value*	HR (95%CI)	*P-value*
Healthy weight	**1.00 (Reference)**		**1.00 (Reference)**	
Underweight	1.63 (1.42 ~ 1.88)	<.001	1.44 (1.25 ~ 1.66)	<.001
Overweight	0.69 (0.64 ~ 0.74)	<.001	0.72 (0.67 ~ 0.78)	<.001
Obesity	0.69 (0.64 ~ 0.74)	<.001	0.65 (0.60 ~ 0.70)	<.001

Note: Model Ⅰ was adjusted for age and gender; Model Ⅱ was adjusted for all variables. HR, hazard ratio; CI, confidence interval.

To explore the impact of varying degrees of obesity, patients were further classified into the following groups: obesity class I (30 ≤ BMI < 35 kg/m²), obesity class II (35 ≤ BMI < 40 kg/m²), obesity class III (40 ≤ BMI < 60 kg/m²), and super-obesity (BMI ≥ 60 kg/m²). The statistical analysis revealed that the mortality rate was lowest in the obesity class I group (Table 4), while it was notably higher in the super-obesity group. Significant differences in mortality rates were observed across these groups.

**Table 4:** Outcomes of patients based on obesity degrees.

Outcomes	30 ≤ BMI < 35 (n = 4021)	35 ≤ BMI < 40 (n = 1847)	BMI ≥ 40 (n = 1481)	*P-value*
90-day mortality, n (%)	580 (14.42)	297 (16.08)	232 (15.67)	<.001
180-day mortality, n (%)	656 (16.31)	339 (18.35)	266 (17.96)	<.001
1-year mortality, n (%)	774 (19.25)	382 (20.68)	316 (21.34)	<.001

In the multifactorial Cox regression analysis of 1-year mortality by obesity class (I, II, III), adjusting for all covariates, a survival advantage persisted across different obesity grades compared to normal-weight patients (Table 5). The same result was also reflected in the multivariate Cox regression analysis of the 90-day and 180-day mortality rates (S3 and S4 Tables).

**Table 5:** Outcomes after classifying BMI into obesity classes.

Obesity grade	Cox multivariate analysis	
	HR (95%CI)	*P-value*
Healthy weight	**1.00 (Reference)**	
Obesity grade Ⅰ	0.65 (0.59 ~ 0.71)	<.001
Obesity grade Ⅱ	0.63 (0.56 ~ 0.70)	<.001
Obesity grade Ⅲ	0.62 (0.54 ~ 0.71)	<.001

Obesity grade Ⅰ (30 ≤ BMI < 35 kg/m²)

Obesity grade Ⅱ (35 ≤ BMI < 40 kg/m²)

Obesity grade Ⅲ (BMI ≥ 40 kg/m²)

Specifically, for Obesity Class I (30 ≤ BMI < 35 kg/m²), there was a significant reduction in the risk of death, with a hazard ratio (HR) of 0.65 (95% CI: 0.59–0.71, p < 0.001), indicating a 35% lower risk of death compared to the normal weight group. For Obesity Class II (35 ≤ BMI < 40 kg/m²), the HR was 0.63 (95% CI: 0.56–0.70, p < 0.001), reflecting a 37% reduction in mortality risk. In Obesity Class III (40 kg/m² ≤ BMI), the risk of death was lowest, with an HR of 0.62 (95% CI: 0.54–0.71, p < 0.001), representing a 38% reduction in risk.

**Fig 3:**
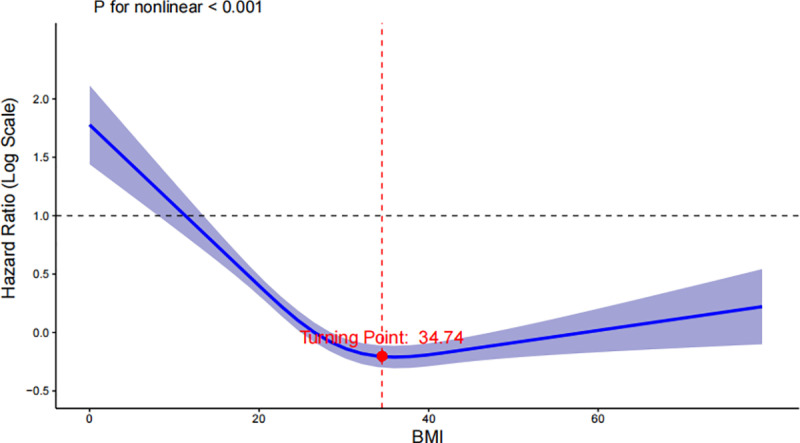
The association between BMI and 1-year mortality in the critically ill patients after adjusting for confounding factors.

A 4-knot restricted cubic spline (RCS) model was constructed to further explore the relationship between BMI and patient mortality (Fig 3). The figure below illustrates the dose-response curves for BMI as a continuous variable and all-cause mortality after adjusting for all covariates. The analysis revealed a U-shaped association between BMI and the risk of 1-year all-cause mortality in critically ill patients without malignant tumors (p for nonlinear < 0.001), with a turning point at BMI 34.74. Mortality decreased with increasing BMI, however, beyond the turning point of 34.74, the hazard ratio (HR) begins to rise gradually, suggesting an increased mortality risk with higher BMI levels. Similar correlations were observed for both 90-day and 180-day all-cause mortality. Confidence intervals around the spline further support the robustness of this association.

Propensity score matching (PSM) was employed to control for confounding variables and ensure that patients across BMI subgroups were otherwise similarly characterized, enabling a more accurate assessment of the independent effects of BMI. We stratified patients based on a BMI cut-off of 25 and applied 1:1 matching using PSM (Table 6). Propensity scores were generated using a logistic regression model to balance patients on baseline characteristics between the two BMI groups. A Standardized Mean Difference (SMD) of less than 0.1 between matched groups indicated successful matching. The baseline characteristics of patients in the full cohort and propensity score matched cohort are shown in S5 Table. Further subgroup analyses, based on age, gender, and mechanical ventilation status after PSM, revealed no significant interaction in most stratified variables. BMI continued to demonstrate a protective effect across these subgroups.

**Table 6:** A subgroup analysis for the effect of obesity on the risk of 1-year mortality after adjusting for all confounding factors. Overweight/obesity *vs.* Normal/underweight matched.

Variables	Cox multivariate analysis		
	HR (95%CI)	*P-value*	P for interaction
Overall	0.64 (0.60 ~ 0.69)	<.001	
**Age**			0.491
<65	0.67 (0.58 ~ 0.76)	<.001	
≥65	0.64 (0.58 ~ 0.69)	<.001	
**Gender**			0.051
Female	0.71 (0.63 ~ 0.79)	<.001	
Male	0.60 (0.54 ~ 0.66)	<.001	
**MV**			0.108
No	0.68 (0.61 ~ 0.76)	<.001	
Yes	0.62 (0.56 ~ 0.68)	<.001	
**Hypertension**			0.614
No	0.65 (0.59 ~ 0.72)	<.001	
Yes	0.65 (0.57 ~ 0.73)	<.001	
**T2DM**			0.646
No	0.65 (0.60 ~ 0.71)	<.001	
Yes	0.61 (0.53 ~ 0.71)	<.001	
**CKD**			0.427
No	0.66 (0.61 ~ 0.72)	<.001	
Yes	0.60 (0.51 ~ 0.70)	<.001	
**Glucocorticoids**			0.991
No	0.64 (0.59 ~ 0.70)	<.001	
Yes	0.65 (0.57 ~ 0.75)	<.001	

## Discussion

Our study adds further evidence to the growing body of research supporting the existence of the obesity paradox in critically ill patients. After adjusting for potential confounders and conducting propensity score matching (PSM) alongside multivariate Cox regression analysis, we found that overweight and obese patients exhibited better 90-day,180-day and 1-year survival rates compared to their normal-weight counterparts. These findings remained robust across various sensitivity analyses, including the application of restricted cubic splines (RCS) to account for the non-linear relationship between BMI and mortality. Unlike many previous studies that primarily focused on short-term outcomes such as ICU or in-hospital mortality, our study aimed to provide new insights into long-term prognosis. One-year mortality was chosen as the primary outcome because it allows for a more comprehensive evaluation of BMI’s impact beyond the acute phase of critical illness. Short-term mortality is often influenced by immediate disease severity and intensive care interventions, whereas long-term mortality better reflects the cumulative effects of metabolic health, nutritional status, and post-ICU recovery.

Most existing studies have concluded that obese patients face challenges such as altered pharmacodynamics, difficulty in nutritional support [16, 17], and are associated with the risk of multi-system diseases, such as cardiovascular and endocrine diseases [18]. Nonetheless, in recent years, an increasing number of studies have suggested that overweight or obese patients may have lower mortality rates compared to normal-weight individuals—a phenomenon referred to as the “obesity paradox.” Although the exact mechanisms behind this paradox remain unclear, it has been observed across various conditions, including heart failure, sepsis, atrial fibrillation, post-coronary revascularization, peripheral artery disease, and chronic critical illness [9, 10, 12, 19–20]. Consistent with our findings, a prior study demonstrated that overweight and obese patients with sepsis in the ICU had lower 30-day and 1-year mortality rates [21]. Similarly, Xu et al. reported an obesity paradox in chronic critical illness, where higher BMI was associated with reduced 90-day mortality [12]. Li et al. further identified a correlation between BMI and 30-day all-cause mortality among critically ill patients, revealing improved survival in obese individuals. However, in contrast to our observations, Li et al. did not observe a protective effect in Class III obese patients (BMI ≥ 40 kg/m²) [22]. This discrepancy may stem from differences in sample sizes, patient populations, and the observation period. Unlike previous studies, our research expands the population to include all critically ill patients admitted to the ICU, with a one-year follow-up, while specifically excluding those with malignancies to minimize confounding effects related to pathological weight loss. We also accounted for the use of glucocorticoids during hospitalization as a covariate in our propensity score matching, to prevent bias in BMI due to glucocorticoid-affected fat redistribution.

Several possible mechanisms may explain the obesity paradox. One theory suggests that adipose tissue provides critical energy reserves during periods of metabolic stress, particularly for patients unable to maintain normal nutritional intake. Additionally, the highly standardized management protocols in the ICU may mitigate the adverse effects of obesity-related comorbidities, such as hypertension and diabetes. Recent studies have also proposed that adipose tissue-derived hormones like leptin may play a cardioprotective role by reducing cardiomyocyte apoptosis [23]. Furthermore, adiponectin, another hormone secreted by adipose tissue, has demonstrated anti-inflammatory effects in preclinical studies. However, the effects of adiponectin in real-world clinical settings appear to vary across patient groups and may differ based on gender [24]. Despite the evidence above, this theory remains controversial, as BMI does not account for factors such as smoking history, alcohol consumption, or the duration of obesity [25]. Moreover, individuals with the same BMI may have vastly different metabolic profiles and cardiopulmonary function due to variations in fat distribution. Most current studies have been conducted in European and American populations, raising the question of whether these findings can be generalized to other regions and ethnicities.

Our subgroup analyses revealed that higher BMI was also associated with better survival outcomes in patients with comorbid hypertension, type 2 diabetes mellitus, and chronic kidney disease, consistent with previous findings. It was previously believed that obese patients requiring mechanical ventilation would experience worse respiratory outcomes due to increased chest wall elasticity and reduced respiratory compliance [26], However, two studies investigating the relationship between BMI and clinical outcomes in ARDS patients found that the obesity paradox may also apply to mechanically ventilated patients with ARDS [27, 28], consistent with our findings.

Despite the non-linear relationship between BMI and mortality, the optimal body weight for critically ill patients is unclear, as this population is highly heterogeneous, even when confounders are carefully adjusted. Future studies focusing on specific etiologies may offer more meaningful insights. Our study identifies a BMI threshold of 34.74, indicating that mortality risk is lowest within the range of Class I obesity. This finding aligns with previous research on the “obesity paradox” in critically ill patients [29, 30]. However, further studies are required to validate the accuracy and generalizability of this result.

Our study has the following limitations: First, being a single-center retrospective study, it is susceptible to selection bias. Although we adjusted for the effects of confounders as much as possible, confounders could not be completely excluded due to database record limitations, such as the use of medications that affect fat distribution, such as glucocorticoid use prior to hospital admission, which could not be extracted from the database. Second, nearly half of the patients were excluded due to missing height or weight data, which may bias the outcome. Third, BMI is an indirect measure of obesity and does not account for variations in body composition or fat distribution [31], which may influence the accuracy of our results. Fourth, the obesity paradox observed in critically ill patients without malignant tumors in our study indicates association, not causality. Therefore, further large-scale, multicenter prospective studies are necessary to determine whether a causal relationship exists between obesity and outcomes in critically ill patients. Lastly, due to database limitations, the primary causes of death after hospital discharge could not be determined, which may potentially limiting the clinical significance of our findings.

## Conclusion

It was found that overweight and obese patients had better long-term survival rates compared to normal-weight patients, further confirming that the obesity paradox may apply to critically ill patients without malignant tumors. However, further research is needed to confirm these findings and elucidate the underlying mechanisms.

## Supporting information

S1 Table Univariate COX regression results and multivariate COX regression results for all variables. (DOCX)

S2 Table Variance inflation factors for all covariates. (DOCX)

S3 Table Multifactorial Cox regression analysis of 90-day mortality after adjustment for all covariates. (DOCX)

S4 Table Multifactorial Cox regression analysis of 180-day mortality after adjustment for all covariates. (DOCX)

S5 Table Baseline characteristics of patients in the full cohort and propensity score matched cohort. (DOCX)
