# Supplementary material for: Association between body mass index and long-term all-cause mortality in critically ill patients without malignant tumors
Source: PLoS One. 2025 Jun 25;20(6):e0325452. doi: 10.1371/journal.pone.0325452 (PMC12193744; doi:10.1371/journal.pone.0325452)
Supplement: S1 Table — (DOCX) [file pone.0325452.s001.docx]

**S1 Table. Univariate COX regression results and multivariate COX regression results for all variables.**

| Variables | *P* | Cox univariate analysis | | | *P* | | Cox multivariate analysis | | | |  |
| --- | --- | --- | --- | --- | --- | --- | --- | --- | --- | --- | --- |
|  |  | OR (95%CI) | | |  |  | OR (95%CI) | | | |  |
| BMI levels |  |  | |  | | | | |  |  |  |
| Healthy weight |  | 1.00 (Reference) | |  | | | | | 1.00 (Reference) |  |  |
| Underweight | <.001 | 1.88 (1.56 ~ 2.26) | | <.001 | | | | | 1.66 (1.33 ~ 2.06) |  |  |
| Overweight | <.001 | 0.63 (0.57 ~ 0.68) | | <.001 | | | | | 0.63 (0.57 ~ 0.70) |  |  |
| Obesity | <.001 | 0.57 (0.52 ~ 0.62) | | <.001 | | | | | 0.54 (0.48 ~ 0.59) |  |  |
| Gender | <.001 | | 0.77 (0.72 ~ 0.83) | | | 0.499 | | 0.97 (0.89 ~ 1.06) | | | |
| MV | 0.030 | | 1.08 (1.01 ~ 1.15) | | | 0.796 | | 0.99 (0.90 ~ 1.09) | | | |
| RRT | <.001 | | 6.14 (5.37 ~ 7.01) | | | <.001 | | 1.54 (1.30 ~ 1.83) | | | |
| Hypertension | <.001 | | 0.64 (0.60 ~ 0.69) | | | 0.002 | | 0.86 (0.78 ~ 0.95) | | | |
| T2DM | <.001 | | 1.31 (1.21 ~ 1.40) | | | <.001 | | 0.72 (0.65 ~ 0.79) | | | |
| HF | <.001 | | 2.32 (2.16 ~ 2.49) | | | 0.004 | | 0.87 (0.79 ~ 0.95) | | | |
| MI | <.001 | | 1.38 (1.23 ~ 1.54) | | | <.001 | | 0.70 (0.61 ~ 0.81) | | | |
| CKD | <.001 | | 2.29 (2.11 ~ 2.49) | | | <.001 | | 0.56 (0.49 ~ 0.63) | | | |
| ARF | <.001 | | 3.88 (3.62 ~ 4.16) | | | <.001 | | 1.55 (1.41 ~ 1.70) | | | |
| Glucocorticoids | <.001 | | 2.06 (1.91 ~ 2.23) | | | <.001 | | 1.22 (1.11 ~ 1.34) | | | |
| Age | <.001 | | 1.04 (1.03 ~ 1.04) | | | <.001 | | 1.01 (1.01 ~ 1.02) | | | |
| SOFA | <.001 | | 1.19 (1.18 ~ 1.20) | | | 0.013 | | 1.02 (1.01 ~ 1.04) | | | |
| APSIII | <.001 | | 1.04 (1.04 ~ 1.04) | | | <.001 | | 1.03 (1.03 ~ 1.04) | | | |
| SAPSII | <.001 | | 1.06 (1.06 ~ 1.06) | | | 0.291 | | 1.00 (0.99 ~ 1.00) | | | |
| Oasis | <.001 | | 1.08 (1.08 ~ 1.09) | | | <.001 | | 1.02 (1.01 ~ 1.03) | | | |
| GCS | <.001 | | 0.96 (0.95 ~ 0.97) | | | <.001 | | 1.10 (1.08 ~ 1.12) | | | |
| Charlson | <.001 | | 1.37 (1.35 ~ 1.39) | | | <.001 | | 1.33 (1.29 ~ 1.36) | | | |
| HR | <.001 | | 1.01 (1.01 ~ 1.01) | | | 0.054 | | 1.00 (1.00 ~ 1.00) | | | |
| MBP | 0.324 | | 1.00 (1.00 ~ 1.00) | | |  | |  | | | |
| RR | <.001 | | 1.07 (1.06 ~ 1.07) | | | <.001 | | 1.02 (1.02 ~ 1.03) | | | |
| SPO_2_ | 0.559 | | 1.00 (1.00 ~ 1.00) | | |  | |  | | | |
| Temperature | <.001 | | 0.97 (0.96 ~ 0.98) | | | 0.004 | | 0.98 (0.96 ~ 0.99) | | | |
| WBC | <.001 | | 1.02 (1.01 ~ 1.02) | | | 0.466 | | 1.00 (1.00 ~ 1.01) | | | |
| Platele | <.001 | | 1.01 (1.01 ~ 1.01) | | | 0.016 | | 1.01 (1.01 ~ 1.01) | | | |
| Hemoglobin | <.001 | | 0.95 (0.93 ~ 0.96) | | | 0.070 | | 1.02 (1.00 ~ 1.04) | | | |
| Sodium | <.001 | | 0.99 (0.98 ~ 0.99) | | | <.001 | | 1.06 (1.05 ~ 1.08) | | | |
| Potassium | <.001 | | 1.16 (1.10 ~ 1.21) | | | 0.006 | | 0.92 (0.87 ~ 0.98) | | | |
| Calcium | 0.617 | | 0.99 (0.95 ~ 1.03) | | |  | |  | | | |
| Chloride | <.001 | | 0.94 (0.93 ~ 0.94) | | | <.001 | | 0.93 (0.92 ~ 0.94) | | | |
| Anion Gap | <.001 | | 1.14 (1.13 ~ 1.14) | | | 0.162 | | 1.01 (1.00 ~ 1.02) | | | |
| pH | <.001 | | 0.04 (0.02 ~ 0.05) | | | 0.028 | | 0.58 (0.35 ~ 0.94) | | | |
| Creatinine | <.001 | | 1.32 (1.29 ~ 1.35) | | | <.001 | | 0.94 (0.91 ~ 0.97) | | | |
