# Supplementary material for: Association between body mass index and long-term all-cause mortality in critically ill patients without malignant tumors
Source: PLoS One. 2025 Jun 25;20(6):e0325452. doi: 10.1371/journal.pone.0325452 (PMC12193744; doi:10.1371/journal.pone.0325452)
Supplement: S2 Table — (DOCX) [file pone.0325452.s002.docx]

**S2 Table. Variance inflation factors for all covariates.**

| Variables | VIF |
| --- | --- |
| Gender | 1.134 |
| Mechanical Ventilation | 1.523 |
| RRT | 1.319 |
| Hypertension | 1.398 |
| T2DM | 1.283 |
| CHF | 1.357 |
| MI | 1.057 |
| CKD | 1.764 |
| ARF | 1.503 |
| Glucocorticoids | 1.095 |
| Age | 2.291 |
| SOFA | 2.698 |
| APSIII | 3.806 |
| Oasis | 2.431 |
| GCS | 1.816 |
| Charlson | 3.300 |
| HR | 1.277 |
| RR | 1.265 |
| Temperature | 1.017 |
| WBC | 1.122 |
| Platele | 1.307 |
| Hemoglobin | 1.295 |
| Sodium | 2.223 |
| Potassium | 1.216 |
| Chloride | 2.824 |
| Anion Gap | 1.914 |
| pH | 1.326 |
| Creatinine | 1.614 |
