# Supplementary material for: Association between body mass index and long-term all-cause mortality in critically ill patients without malignant tumors
Source: PLoS One. 2025 Jun 25;20(6):e0325452. doi: 10.1371/journal.pone.0325452 (PMC12193744; doi:10.1371/journal.pone.0325452)
Supplement: S3 Table — (DOCX) [file pone.0325452.s003.docx]

**S3 Table.** **Multifactorial Cox regression analysis of 90-day mortality after adjustment for all covariates.**

| Variables |  | Cox multivariate analysis | |
| --- | --- | --- | --- |
|  |  | HR (95%CI) | *P* |
| BMI levels |  |  |  |
| Healthy weight |  | **1.00 (Reference)** |  |
| Underweight |  | 1.38 (1.17 ~ 1.62) | <.001 |
| Overweight |  | 0.73 (0.67 ~ 0.79) | <.001 |
| Obesity |  | 0.63 (0.58 ~ 0.69) | <.001 |
