# Supplementary material for: Association between body mass index and long-term all-cause mortality in critically ill patients without malignant tumors
Source: PLoS One. 2025 Jun 25;20(6):e0325452. doi: 10.1371/journal.pone.0325452 (PMC12193744; doi:10.1371/journal.pone.0325452)
Supplement: S5 Table — (DOCX) [file pone.0325452.s005.docx]

**S5 Table. Baseline characteristics of patients in the full cohort and propensity score matched cohort.**

| Variable | Before PSM | | | | | | After PSM | | |
| --- | --- | --- | --- | --- | --- | --- | --- | --- | --- |
|  | Total  (n = 19089) | BMI < 25  (n = 5486) | BMI≥25  (n = 13603) | SMD |  | Total  (n = 10958) | BMI < 25  (n = 5479) | BMI≥25  (n = 5479) | SMD |
| Age, years | 66 (55, 76) | 68 (54, 80) | 65.00 (55, 75) | -0.095 |  | 67 (55, 78) | 68 (54, 80) | 67 (56, 76) | -0.029 |
| Gender, n (%) | 11622 (60.88) | 3048 (55.56) | 8574 (63.03) | 0.155 |  | 6248 (57.02) | 3045 (55.58) | 3203 (58.46) | 0.059 |
| **Vital signs** |  |  |  |  |  |  |  |  |  |
| HR | 84 (74, 98) | 85 (74, 100) | 83 (74, 97) | -0.064 |  | 85 (74, 99) | 85 (74, 100) | 84 (74, 98) | -0.028 |
| MBP | 78 (68, 90) | 79 (68, 91) | 77 (68, 90) | 0.010 |  | 78 (68, 91) | 79 (68, 91) | 78 (68, 90) | 0.012 |
| RR | 17 (14, 22) | 18 (14, 22) | 17 (14, 21) | -0.095 |  | 17 (14, 22) | 18 (14, 22) | 17 (14, 22) | -0.039 |
| SPO2 | 99 (96, 100) | 99 (96, 100) | 99 (96, 100) | 0.008 |  | 99 (96, 100) | 99 (96, 100) | 99 (96, 100) | -0.005 |
| Temperature | 36.7 (36.4, 37.0) | 36.7 (36.3, 37.0) | 36.7 (36.4, 37.0) | 0.046 |  | 36.7 (36.4, 37.0) | 36.7 (36.3, 37.0) | 36.7 (36.4, 37.0) | 0.030 |
| **Laboratory tests** |  |  |  |  |  |  |  |  |  |
| WBC | 11.4 (8.2, 15.5) | 10.7 (7.6, 14.7) | 11.7 (8.5, 15.8) | 0.087 |  | 11.0 (7.9, 15.1) | 10.7 (7.6, 14.7) | 11.3 (8.2, 15.5) | 0.045 |
| Platele | 177 (130, 237) | 184 (129, 250) | 175 (130, 233) | -0.130 |  | 182 (130, 245) | 184 (129, 250) | 180 (132, 240) | -0.059 |
| Hemoglobin | 10.4 (8.9, 12.0) | 10.3 (8.8, 11.9) | 10.5 (9.0, 12.1) | 0.089 |  | 10.4 (8.8, 11.9) | 10.3 (8.8, 11.9) | 10.4 (8.8, 12.0) | 0.028 |
| Sodium | 139 (136, 141) | 139 (136, 141) | 139 (136, 141) | 0.005 |  | 139 (136, 141) | 139 (136, 141) | 139 (136, 141) | 0.004 |
| Potassium | 4.2 (3.8, 4.6) | 4.1 (3.7, 4.5) | 4.2 (3.8, 4.6) | 0.185 |  | 4.1 (3.7, 4.5) | 4.1 (3.7, 4.5) | 4.1 (3.8, 4.5) | 0.080 |
| Calcium | 8.29 (7.90, 8.70) | 8.29 (7.80, 8.70) | 8.29 (7.90, 8.70) | 0.057 |  | 8.29 (7.80, 8.70) | 8.29 (7.80, 8.70) | 8.30 (7.90, 8.70) | 0.027 |
| Chloride | 106 (102, 109) | 105 (101, 109) | 106 (102, 109) | 0.038 |  | 105 (101, 109) | 105 (101, 109) | 106 (102, 109) | 0.011 |
| Anion Gap | 13 (11, 16) | 14 (11, 16) | 13 (11, 16) | -0.035 |  | 14 (11, 16) | 14 (11, 16) | 13 (11, 16) | -0.014 |
| pH | 7.37 (7.34, 7.42) | 7.37 (7.35, 7.43) | 7.37 (7.34, 7.42) | -0.079 |  | 7.37 (7.35, 7.42) | 7.37 (7.35, 7.43) | 7.37 (7.34, 7.42) | -0.048 |
| Creatinine | 0.9 (0.7, 1.3) | 0.9 (0.7, 1.3) | 1.0 (0.7, 1.4) | 0.085 |  | 0.9 (0.7, 1.3) | 0.9 (0.7, 1.3) | 0.9 (0.7, 1.3) | 0.048 |
| **Scoring system** |  |  |  |  |  |  |  |  |  |
| SOFA | 5 (2, 7) | 4 (2, 7) | 5 (3, 8) | 0.135 |  | 4 (2, 7) | 4 (2, 7) | 4 (2, 7) | 0.062 |
| APSIII | 40 (29, 56) | 42 (30, 56) | 39 (29, 55) | -0.054 |  | 40 (30, 56) | 42 (30, 56) | 39 (29, 56) | -0.029 |
| SAPSII | 35 (28, 45) | 35 (28, 45) | 35 (28, 45) | 0.003 |  | 35 (28, 45) | 35 (28, 45) | 36 (28, 45) | 0.008 |
| Oasis | 32 (27, 38) | 32 (27, 39) | 32 (27, 38) | -0.036 |  | 32 (27, 38) | 32 (27, 39) | 32 (27, 38) | -0.014 |
| GCS | 15 (14, 15) | 15 (14, 15) | 15 (14, 15) | 0.058 |  | 15 (14, 15) | 15 (14, 15) | 15 (14, 15) | 0.026 |
| Charlson | 4 (2, 6) | 5 (2, 6) | 4 (2, 6) | -0.060 |  | 4 (3, 6) | 5 (2, 6) | 4 (3, 6) | -0.015 |
| **Comorbidities, n (%)** |  |  |  |  |  |  |  |  |  |
| Hypertension | 8684 (45.49) | 2076 (37.84) | 6608 (48.58) | 0.215 |  | 4377 (39.94) | 2075 (37.87) | 2302 (42.01) | 0.084 |
| T2DM | 5387 (28.22) | 963 (17.55) | 4424 (32.52) | 0.320 |  | 2222 (20.28) | 963 (17.58) | 1259 (22.98) | 0.128 |
| CHF | 5111 (26.77) | 1409 (25.68) | 3702 (27.21) | 0.034 |  | 2872 (26.21) | 1409 (25.72) | 1463 (26.70) | 0.022 |
| MI | 1660 (8.7) | 439 (8.00) | 1221 (8.98) | 0.034 |  | 889 (8.11) | 439 (8.01) | 450 (8.21) | 0.007 |
| CKD | 3082 (16.15) | 796 (14.51) | 2286 (16.81) | 0.061 |  | 1654 (15.09) | 796 (14.53) | 858 (15.66) | 0.031 |
| ARF | 5685 (29.78) | 1474 (26.87) | 4211 (30.96) | 0.088 |  | 3064 (27.96) | 1474 (26.90) | 1590 (29.02) | 0.047 |
| **Interventions, n(%)** |  |  |  |  |  |  |  |  |  |
| Ventilation | 11311 (59.25) | 2963 (54.01) | 8348 (61.37) | 0.151 |  | 6059 (55.29) | 2960 (54.02) | 3099 (56.56) | 0.051 |
| RRT | 1007 (5.28) | 195 (3.55) | 812 (5.97) | 0.102 |  | 444 (4.05) | 195 (3.56) | 249 (4.54) | 0.047 |
| Glucocorticoids | 3815 (19.99) | 1276 (23.26) | 2539 (18.67) | -0.118 |  | 2451 (22.37) | 1272 (23.22) | 1179 (21.52) | -0.041 |
